# *Plasmodium vivax* Tryptophan-Rich Antigen PvTRAg33.5 Contains Alpha Helical Structure and Multidomain Architecture

**DOI:** 10.1371/journal.pone.0016294

**Published:** 2011-01-20

**Authors:** Hema Bora, Sheena Garg, Priyankar Sen, Deepak Kumar, Punit Kaur, Rizwan Hasan Khan, Yagya D. Sharma

**Affiliations:** 1 Department of Biotechnology, All India Institute of Medical Sciences, New Delhi, India; 2 Interdisciplinary Biotechnology Unit, Aligarh Muslim University, Aligarh, India; 3 Department of Biophysics, All India Institute of Medical Sciences, New Delhi, India; Université Pierre et Marie Curie, France

## Abstract

Tryptophan-rich proteins from several malarial parasites have been identified where they play an important role in host-parasite interaction. Structural characterization of these proteins is needed to develop them as therapeutic targets. Here, we describe a novel *Plasmodium vivax* tryptophan-rich protein named PvTRAg33.5. It is expressed by blood stage(s) of the parasite and its gene contains two exons. The exon 1 encodes for a 23 amino acids long putative signal peptide which is likely to be cleaved off whereas the exon 2 encodes for the mature protein of 252 amino acids. The mature protein contains B-cell epitopes which were recognized by the human immune system during *P.vivax* infection. The PvTRAg33.5 contains 24 (9.5%) tryptophan residues and six motifs whose patterns were similar among tryptophan-rich proteins. The modeled structure of the PvTRAg33.5 consists of a multidomain architecture which is stabilized by the presence of large number of tryptophan residues. The recombinant PvTRAg33.5 showed predominantly α helical structure and alpha helix to beta sheet transition at pH below 4.5. Protein acquires an irreversible non-native state at temperature more than 50°C at neutral pH. Its secondary and tertiary structures remain stable in the presence of 35% alcohol but these structures are destabilized at higher alcohol concentrations due to the disturbance of hydrophobic interactions between tryptophanyl residues. These structural changes in the protein might occur during its translocation to interact with other proteins at its final destination for biological function such as erythrocyte invasion.

## Introduction

Malaria remains uncontrolled to date and requires effective antimalarial drugs and vaccines [1]. Both, vaccine and drug development strategies require identification and characterization of the target molecules. The parasite molecules which interact with the host molecules are good drug or vaccine targets. This is because any molecule or antibody which can disrupt this host - parasite molecular interaction can be used as therapeutic reagent to interrupt the disease progression. Enormous efforts are therefore being made to identify such parasite molecules. However, in comparison to the *Plasmodium falciparum*, only fewer vaccine/drug target molecules of *P.vivax* have been identified because of its non-cultivable nature although this parasite affects large number of people in tropical countries and sometime can cause severe complications in humans [1], [2], [3].

Tryptophan-rich proteins from several *Plasmodium* species have been identified. They play an important role in the host-parasite interaction [4], [5], [6], [7], [8]. Some of them may be involved in reinvasion of the host erythrocytes by the parasite [5]. The synthetic peptides derived from the *P.falciparum* tryptophan-threonine rich antigen (PfTryThrA) were able to inhibit the invasion of the erythrocyte by the merozoite [9]. These proteins across the malarial species contain positional conservation of the tryptophan residues which could be functionally important for the parasite. The *P. vivax* genome encodes for more number of such tryptophan-rich proteins than any other malarial species (http://www.plasmodb.org). These proteins need to be characterized so as to develop them as the drug or vaccine targets. Recently, we have described immunological characterization of some of these *P.vivax* proteins and reported that they were highly immunogenic in humans [10], [11], [12], [13], [14]. The tryptophan-rich domains of all these proteins were also conserved in the parasite population. Unfortunately, so far none of the tryptophan - rich antigen of any of the *Plasmodium* species has been structurally characterized. Here, we describe cloning, expression, human humoral immune response, physico-chemical characterization and molecular modeling of a novel 33.5 kDa *P. vivax* tryptophan-rich protein called PvTRAg33.5 which showed a significant homology to the PfTryThrA of *P.falciparum*.

## Materials and Methods

### Reverse transcription PCR

Previously isolated RNA from the *P.vivax -* infected blood was used for a two step reverse transcription PCR with oligodT primers and random decamers using RETROScript® (Ambion, Inc. Austin, TX USA) as per the manufacturer's instructions. The exon 1 and part of the exon 2 was amplified from the cDNA as well as the genomic DNA (to detect intron) using the gene specific primers F1 (forward) (5′-ATGGTTGCCTTATTACCAATTTCA-3′) and R1 (reverse) (5′-AGTTTTCCCAGTCAGCATCATTCC-3′). A 2 µL of 1∶5 dilution of the primary PCR product was used as a template for the semi-nested PCR using primers F2 (5′-TCTGCGGCTTACCTTTTAAGCAAC-3′) and R1 (mentioned above). A 50 µL PCR reaction mixture included the Taq DNA polymerase (1U) with 1X PCR buffer, 0.5 mM dNTPs, 1.5 mM MgCl_2_, 0.5 µM of each primer and 10 ng of the template DNA. A control reaction was also included in which no reverse transcriptase was added to rule out the possibility of the DNA contamination after DNase treatment in the cDNA synthesis. The PCR cycling conditions included initial denaturation at 94°C for 5 min followed by 35 cycles of denaturation at 94°C for 30 sec, annealing at 56°C (for F1 and R1) or 58°C (for F2 and R1) for 30 sec and extension at 72°C for 30 sec. Final extension was carried out at 72°C for 5 minutes. The amplified products were purified from the gel using AccuPrep® gel purification kit (Bioneer Corporation, korea) and sequenced from both the strands using F2 and R1 primers on the ABI Prism A310 Genetic Analyzer (PE Applied Biosystems, CA, USA) as described before [15].

### Cloning, expression and purification of the recombinant PvTRAg33.5

The exon 2 of *pvtrag33.5* gene was PCR amplified from the *P.vivax* DNA using primers, 5′-TGTAGTCGACTCAAAGCGCAGTAG-3′ (forward) and 5′TTGTTCCTAATTGAGTCTAGAATTCC3′ (reverse) having *Sal*I and *Xba*I sites (underlined) respectively. The cycling parameters for PCR included initial denaturation at 94°C for 5 minutes followed by 35 cycles of denaturation at 94°C for 15 sec, annealing at 55°C for 30 sec, extension at 68°C for 1 minute and final extension at 68°C for 15 minutes. The PCR product was cloned into the pGEMT vector and sequenced with the universal primers. The fragment was transferred to the expression vector pPROExHT b (Invitrogen Life Technologies, Carlsbad, CA, USA). The recombinant PvTRAg 33.5 was expressed in *E.coli* BL21 (DE3) codon plus-RP and induced with 1 mM isopropyl-β-D-thiogalactopyranoside (IPTG) at 22°C for 4 hours.

The recombinant protein from the culture pellet was purified using the Immobilized Metal Affinity Chromatography on the Ni^2+^ NTA agarose column according to the manufacturer's instructions (Qiagen, GmbH, Hilden, Germany). It was further purified by the Anion Exchange High Performance Liquid Chromatography (Biologic Duo flow, Biorad laboratories, Hercules, CA, USA). The dialyzed Ni^2+^ NTA purified protein was injected in 50 µl sample loop from where it was loaded on to the top of 1.3 ml UNO Q1 Anion exchange column (Biorad laboratories, Hercules, CA, USA) which had been pre-equilibrated at a flow rate of 2 ml/minute with ten column volumes of buffer A (20 mM Tris, pH 8.0). The bound proteins were then eluted by the NaCl gradient (0–500 mM) by employing buffer B (20 mM Tris pH 8.0 and 0.5 M NaCl) at a flow rate of 2 ml/minute over a period of ten minutes. One ml fractions each were collected and detected at a wavelength of 280 nm by the deuterium lamp in the Quad Tec UV-Vis detector integrated with the HPLC unit. The homogeneity of the purified recombinant PvTRAg33.5 protein was confirmed by the SDS-PAGE as described earlier [15].

### Direct binding Enzyme-Linked Immunosorbent Assay (ELISA)

The ELISA was performed as described earlier [15], [16] using 50 *P. vivax*-infected and 39 uninfected sera samples at a 1∶100 dilution and 100 ng of the purified recombinant PvTRAg33.5 protein. The goat anti-human IgG horseraddish peroxidase conjugate at a 1∶400 dilution (Pierce Chemical Company, Rockford, IL, USA) was used as secondary antibody. The mean OD plus 3 standard deviations of the normal healthy controls was used as the cutoff value for a positive response.

### Physico-chemical characterization of recombinant PvTRAg33.5

All the measurements were carried out at the room temperature, unless stated. The concentration of protein samples were determined spectrophotometrically from the extinction coefficient reported at 280 nm.

#### Circular dichroism measurements

The circular dichroism (CD) was measured with a JASCO J-815 spectropolarimeter calibrated with ammonium d-10 camphorsulfonate. A cell of path length 0.1 cm was used for scanning between 250–195 nm and a cell of path length 1.0 cm was used for scanning between 300–250 nm. The results were expressed as the mean residue ellipticity (MRE) in deg.cm^2^.dmol^−1^, which is defined as:
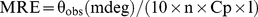
(i)


Where θ_obs_, is the observed ellipticity in degrees, n is the number of peptide bonds per molecule, Cp is the molar concentration, and ‘l’ is the length of light path in cm. The α-helical content of proteins was calculated from the MRE value at 222 nm (MRE_222_) using the following equation [17]:
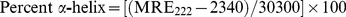
(ii)


CD data have also been analyzed by online available software, K2d [18].

#### Fluorescence measurements

The fluorescence spectra were recorded with a Hitachi F-3500 spectrofluorophotometer in a 1.0 cm path length quartz cell. Samples containing different concentrations of the organic solvent were equilibrated at room temperature for 30 minutes before recording of the fluorescence measurements. The excitation wavelength was 295 nm and the emission was recorded from 300 to 400 nm. The ANS (8-anilino-1-naphthalene-sulfonate) binding was measured by fluorescence emission with excitation at 380 nm and emission was recorded from 400 to 600 nm. Typically, the ANS concentration was 50 times excess of protein concentration (7 µM).

### Sequence analysis

The PvTRAg33.5 full length sequence was analyzed using signal prediction servers, Ipsort [19], and SOSUI [20] utilizing different prediction methods to determine the presence of the signal peptide region. The transmembrane helical region analysis of the PvTRAg33.5 (exon2) was carried out using TMHMM [21] and TM Pred [22] servers. InterPro scan and ProScan [23] analysis was carried out for the motif and/or the pattern prediction. The 91 similar sequences obtained from the PlasmoDB database (http://www.plasmodb.org) were input into MEME [24] to the identify conserved domain, aligned in MAST [25] and fed into meta-MEME [26]. These motifs were then combined into a single, motif-based Hidden Markov Model (HMM) and used for searching homologs. The complete sequence of the PvTRAg33.5 was used for the identification of motifs.

### Secondary structure prediction

The prediction servers incorporating different algorithms were used for the secondary structure prediction to minimize the error in prediction (supplementary material S1). The consensus secondary structure was derived employing the probability of the predicted sequence and the type of secondary structure prediction from these methods. This was compared with the data derived from the experimental CD studies.

### Model development and verification

The initial step for the model development is the identification of a structural homology. Threading technique or fold recognition that assign folds to target sequences with very low sequence identity to known structures can also be employed for the template generation. The threading servers utilized to build initial models included PHYRE [27], Fugue [28], PredictProtein [29], SAM-T08 [30] and iTASSER [31], [32], [33]. These servers exploit various machine learning fold recognition techniques like recognizing distant homologues by sequence-structure comparison, profile-profile alignments and protein folding potentials. The final templates so obtained were used to build a homology model with Discovery Studio v1.7 (www.accelrys.com). Energy minimization of generated model was by CHARMm [34] force field, a implicit distance-dependent dielectric constant and a non-bonded atom cut-off 12 angstrom, by the Adopted Basis Newton-Raphson energy minimization. Two thousand steps of energy minimization of r.m.s. gradient of 0.05 Kcal/(mol× Angstrom) was performed.

The model was checked for its accuracy and correctness by the Ramachandran plot to determine the amount of misplaced phi and psi angles. The stereochemical environment, the conformation of the backbone and side chains was analysed using PROCHECK [35].

### Molecular Dynamics

The overall stability of the PvTRAg33.5 model was checked by a molecular dynamics simulation using the CHARMm [34] module on a fully hydrated model. In the first step of energy minimization, the backbone of model was kept flexible. The minimized, hydrated complex was then subjected to a molecular dynamics simulation in three stages. In the first stage, the temperature of the system was raised from 0 to 300 K for 2 pico sec. The system was then equilibrated for 20 sec, and the final production run was carried out over another 100 picoseconds.

### Accession numbers

The nucleotide sequences described in the paper have been deposited in the Genbank under the accession numbers FJ481116 for the genomic DNA and FJ481117 for the cDNA derived from the *pvtrag33.5* gene of the Indian *P.vivax* isolate.

## Results

### Sequence analysis of the *pvtrag33.5* gene

Homology searches for the tryptophan-rich antigen of *P. vivax* identified a number of related protein molecules in the *Plasmodium* database (http://www.plasmodb.org). We selected a protein named here as PvTRAg33.5 which is of 33.5 kDa mol. wt and rich in tryptophan residues. This protein showed maximum homology of 56% (33% identity) with the PvTRAg35.2 of *P.vivax* (Fig. S1). Its sequence homology with the *P.falciparum* and *P.yoelii* tryptophan rich proteins PfTryThrA and PypAg3 was 55% (30% identity over a 43-275 aa overlap) and 51% (29% identity over a 20-270 aa overlap), respectively. Twenty out of 24 tryptophan residues of PvTRAg33.5 were positionally conserved among these proteins. Several tryptophan residues were posionally conserved across the tryptophan-rich proteins of *Plasmodium* species (Fig. S1). The *pvtrag33.5* gene contains two exons of 69 bp (exon-1) and 756 bp (exon-2). The exon 1 encodes for a putative signal peptide which is likely to be cleaved away and thus may not be part of the mature folded protein. Hence, only the exon2 encoded protein was used for further characterization and model determination. The ProScan analysis found one putative glycosylation site (Asn 81) and eight putative phosphorylation sites (Ser 4, Ser 98, Thr 129, Ser 177, Thr 179, Ser 217, Ser 243 and Thr 232). The sequences input to MEME yielded the presence of seven different motifs in the full length sequence (Table 1). The tryptophan-rich proteins having more than 30% identity contained all 7 motifs in the order ‘M7 M3 M6 M1 M4 M5 M2’. A database search using these clusters with MAST package also did not yield any significant match indicating the absence of orthologues for PvTRAg33.5.

**Table 1:** Motifs identified from MEME present in the various *Plasmodium* species.

Motif No	Motif regular expression	Motif in PvTRAg33.5
M1	YKS[ND]ILKKSSTW[DN][DE]S[QE]W[EK]EW[IM]KTEGKE[LF][MLI]	YKNYLLKKSEKWNDADWENWANTEMVAHL
M2	[FY]NEWM[ED]SF[IV]NKWI[KN]EK[QK]WNVW	YSTWRNDFINRWVSEKKWNSI
M3	WKNNEW[KN][NK]W[MK]KKLEx[DE]WKxFN	WKDNEWHNWKLKLEEDWDSFS
M4	WI[QK]WKNxKIx[ES]WL[MS]S[DE]WKxEEDEYWSKW	WNQWQHDKMSSWLSSDWKKVGAMYWDLQ
M5	xKW[KN]ERIN[RK]ExE[EQ]WxNWVKxKENx[YF]	IKWNDRNARENIEWSKWVQNKEYFI
M6	KEK[ED][WL][EN]EW[LI]Kx[ML][EQ]NKWMH[YF]NE	KTDELNGWLNLEENKWNNFSG
M7	VSxLxIxLFLLSSAF	VALLPISFFSLSAAY

### Expression and purification of the recombinant PvTRAg33.5

**Figure 1:**
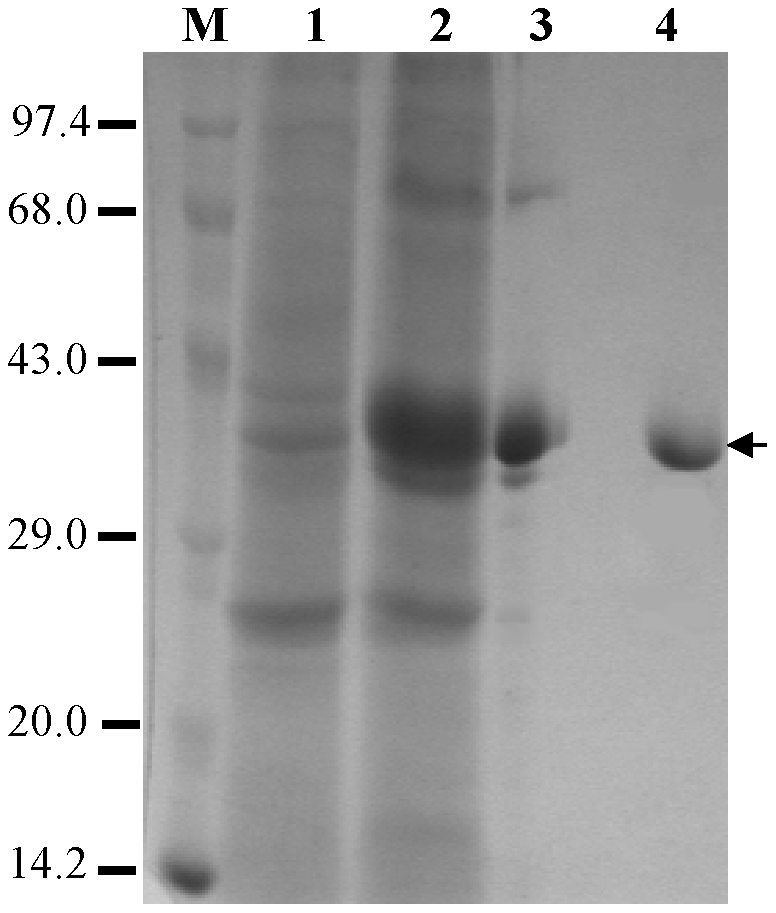
Purification of recombinant PvTRAg33.5. SDS-PAGE profile of the purification steps. Uninduced and induced culture pellets of the recombinant clone were solubilized in the loading buffer and loaded in lane 1 and 2, respectively. The Ni^2+^ NTA column purified preparation was loaded in lane 3. Lane 4 contains the HPLC purified protein. Size of protein bands in the marker lane (M) is indicated on left hand side. The recombinant PvTRAg33.5 band is indicated by an arrow.

We were unable to express the complete cDNA of the *pvtrag 33.5* gene in *E.coli* and thus decided to express the exon 2 coding region and discarded exon 1 as it encodes only for the putative signal peptide which may not be part of the mature protein. The exon 2 encoded protein was found in the inclusion bodies (90%) as well as in the soluble fraction (10%). In order to avoid the in-vitro refolding process, we purified the recombinant protein from the soluble fraction. The recombinant protein was purified on the Ni^2+^ NTA agarose column since the expressed protein contained the His tag. Although the protein was purified to a great extent by this column, the minor contaminants were still present in the preparation (lane3 in Fig. 1). These contaminants were removed by the HPLC using an anion exchange column where this protein eluted as a single peak between 440 and 500 mM NaCl concentration (data not shown). This peak was found to contain only a single band of 33.5 kDa on 12% SDS-PAGE (lane 4 in Fig. 1).

### In vivo expression of the PvTRAg33.5 by *P. vivax*

**Figure 2:**
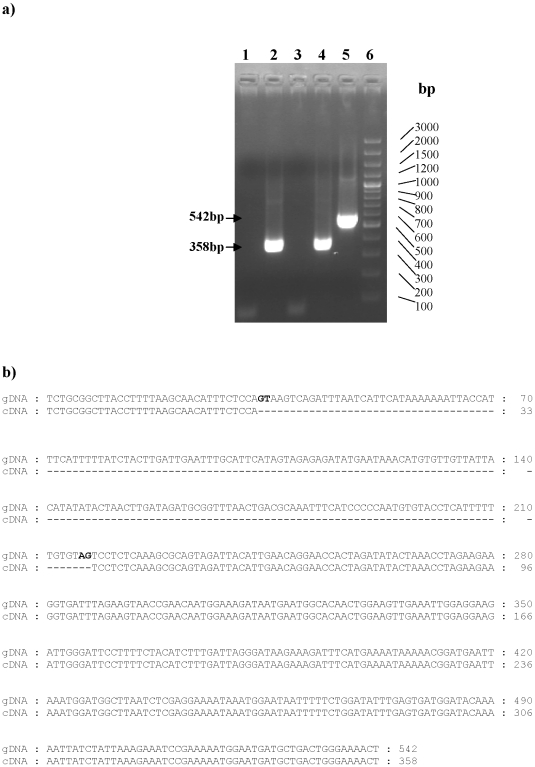
In vivo expression of the *pvtrag33.5* gene. a) Detection of the *pvtrag33.5* gene transcript by Reverse Transcription PCR. PCR amplification of the *P.vivax* cDNA (Lanes 1–4) and genomic DNA (Lane 5) using *pvtrag33.5* gene specific primers. RT- PCR product of pvtrag33.5 specific cDNA synthesized using oligodT (Lane 2) or random decamers (Lane 4) with gene specific primers. Reverse transcription negative control using oligodT (Lane 1) or random decamers (Lane 3) as primers for cDNA synthesis. Lane 5: PCR product of genomic DNA amplification, and Lane 6: 100 bp ladder. PCR product sizes are indicated by arrows. b) Sequence alignment of PvTRAg33.5 genomic DNA and cDNA. Splice site sequence is shown in bold letters. Dashes indicate the absence of nucleotides. Numbers on the right-hand side indicate the number of nucleotides.

Since the *pvtrag33.5* gene was not annotated in the PlasmoDB sequence database at the time of initiating this work, we wanted to know if this gene was actively expressed by the parasite. The RT-PCR results revealed the presence of the *pvtrag33.5* gene transcript in the parasite (Fig. 2a). The size difference between the cDNA and genomic DNA PCR products not only confirmed the specificity of the RT-PCR but also established the presence of an intron in the gene (Fig. 2a). The sequence analysis of the RT-PCR product and its comparison with the genomic DNA sequence revealed that the size of the intron was 184 bp and the consensus splice site sequence GU/AG at the intron-exon junction (Fig. 2b). That this *pvtrag33.5* gene transcript was translated in to the protein product in the parasite was evident from the fact that majority of the *P.vivax* patients produced antibodies against PvTRAg33.5 during the acute phase of infection (described below).

### Humoral immune response against the exon-2 encoded polypeptide of PvTRAg33.5 among a *P.vivax* infected individuals

**Figure 3:**
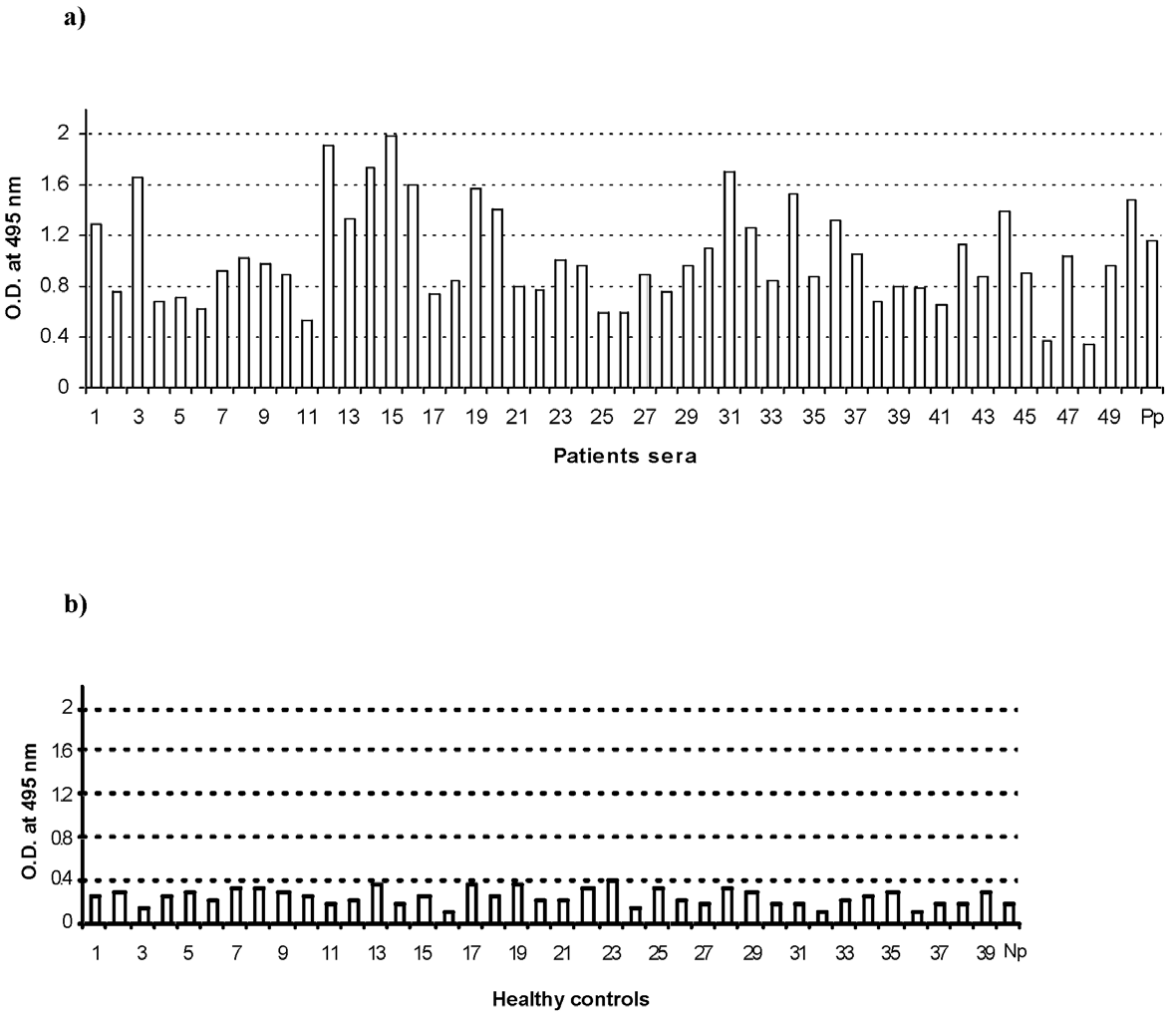
Seroreactivity of recombinant PvTRAg33.5 among *P.vivax* exposed individuals. ELISA was performed with 50 sera form *P. vivax* patients (a) and 39 from unexposed healthy controls (b) by coating 96-well microtitre ELISA plates with the purified recombinant PvTRAg33.5. Each bar represents the OD value for the individual serum. Pp: pooled patient sera; Np: pooled sera from healthy controls.

The natural humoral immune response to the PvTRAg33.5 was confirmed by assessing the reactivity of the exon-2 derived recombinant protein with the antibodies present in each sera sample by the ELISA. The positive responders were defined as those who exhibited an OD_495_ higher than 0.55 (mean +3 SD of control sera). Forty seven of the 50 serum samples (seropositivity 94%) from the *P.vivax* infected individuals gave an OD above this cutoff (Fig. 3). The mean ± SD values of OD for the *P.vivax* infected and naive individuals were 1.04±0.39 and 0.25±0.10, respectively. All individuals had different levels of serum antibodies to the PvTRAg33.5.

### Physico-chemical characterization of the PvTRAg33.5

#### Effect of pH

**Figure 4:**
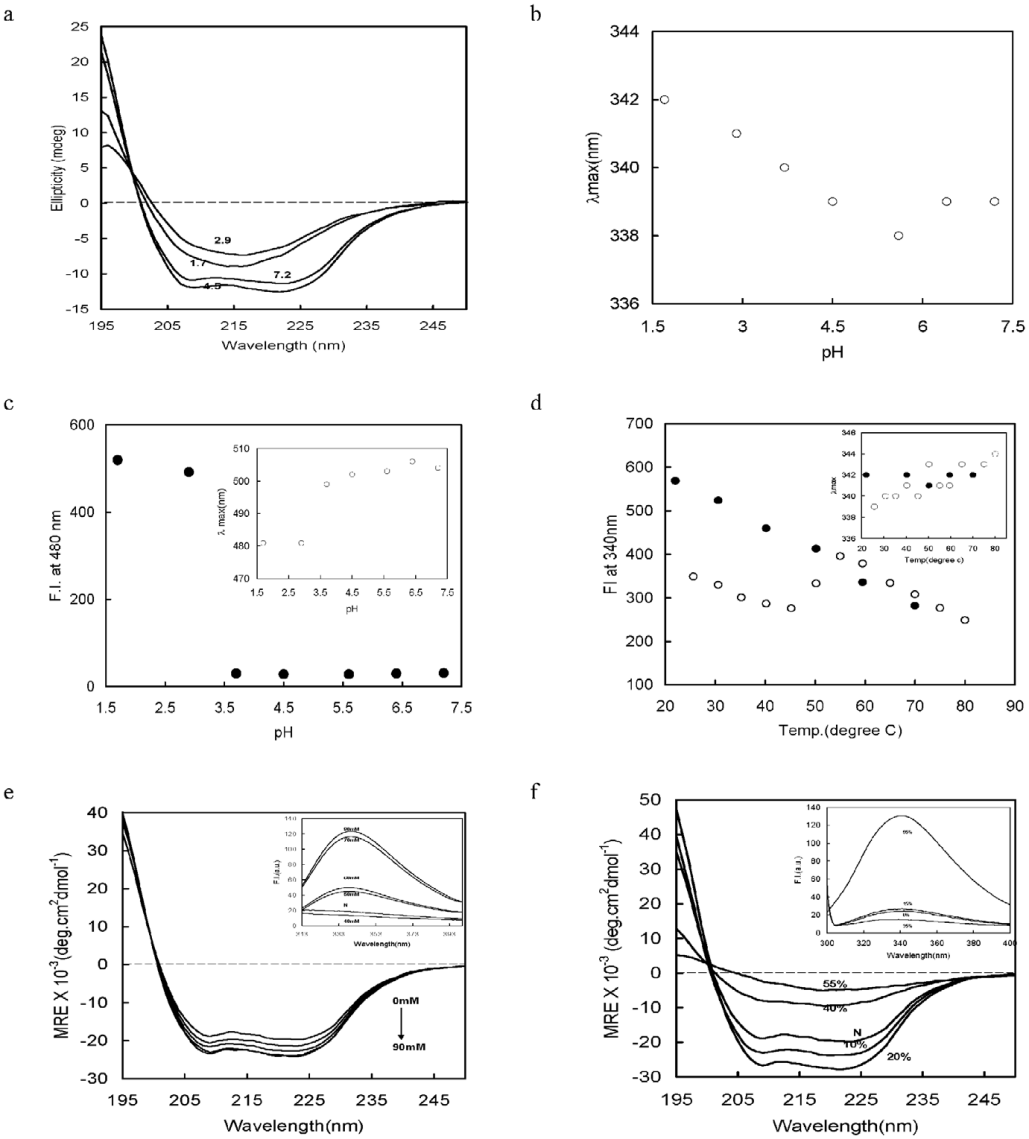
Physico-chemical characterization of PvTRAg33.5. Far UV CD spectra at different pH (a), emission wavelength at maximum intensity after exciting it at 295 nm as a function of pH (b), fluorescence intensity (filled circle) and emission wavelength at maximum intensity (empty circle) (inset) of ANS-protein complex as a function of pH, after exciting it at 380 nm (c), fluorescence intensities and emission wavelength maxima (inset) after exciting at 295 nm as a function of increase in temperature (empty circle) and consecutive decrease in temperature (filled circle) at pH 7.0 (d), far UV CD spectra and fluorescence intensities (inset) in the absence and presence of different ammonium sulfate concentrations at pH 7.0 after excited at 295 nm (e), and far UV CD spectra and fluorescence intensities (inset) in the absence (N) and presence of different ethanol concentrations after exciting at 295 nm (f) of PvTRAg33.5.

Studies of the far UV CD can be used to quantitatively assess the overall secondary structure content of the protein, as the absorbing group is principally the peptide bond. Figure 4a shows the far UV CD of the PvTRAg33.5 at neutral to low pH. The UV CD spectrum at pH 7.2 shows minima between 208 and 222 nm, a characteristic feature of the α-helical proteins [36]. Interestingly, the protein did not show any loss of the secondary structure till pH 4.5, but further decrease in pH resulted in the alpha helix to beta sheet transition with a single negative minima near 215 nm and a positive ellipticity at 197 nm [37]. Further, the formation of an isobestic point at 197 nm also indicates the same [38]. Similar results were also observed with increased ammonium sulfate (see section 3.5.3) and ethanol (section 3.5.4) concentrations.

The tryptophan residues excite exclusively above 295 nm and emit in the range of 300 to 400 nm [39]. Figure 4b shows the shift in wavelength of maximum intensity (λ_max_) as a probe for the tertiary structure of the protein against pH. It shows an increase in λ_max_ as pH decreases, indicating an increase in the polarity of the tryptophan microenvironment, and thus increases in solvent accessibility to the core of the protein.

The hydrophobic dye, 1-anilino-8-nephthalene sulphonate (ANS), is used as a probe to determine the concentration of solvent accessible hydrophobic patches [40]. The fluorescence of protein-ANS complex showed no significant change as pH decreased from 7.3 to 3.6, but it was followed by an increase in the ANS binding with decrease in λ_max_ (Fig. 4c; inset) as pH decreases from 3.6 to 1.6 (Fig. 4c). The results show that the PvTRAg33.5 has lost its secondary and tertiary contacts to a significant level below pH 3.6.

#### Effect of temperature

To understand the stability of the tertiary structure of PvTRAg33.5, we have observed change in tryptophanyl fluorescence maxima (at 340 nm) as a probe for tertiary structure, against increase in temperature (Fig. 4d). The fluorescence intensity has decreased with increase in temperature from 20–45°C, followed by a sudden increase up to 55°C, and then a decrease till 80°C. But on cooling, the increase in fluorescence intensity did not crawled back in the same track below 50°C, indicating the protein might have been trapped in some non-native conformation. The change in λ_max_ seems to support the fact that on heating the λ_max_ increased from 339 to 344 nm as temperature increased from 20 to 80°C, showing increased accessibility of the hydrophobic tryptophan residues in solvent due to unfolding. But on reversing the temperature below 80°C, no significant change was observed, indicating entrapment of the protein molecules in local minima of folding.

#### Effect of ammonium sulfate

Effect of ammonium sulfate (0–90 mM) on PvTRAg33.5 has been observed by the far UV CD and the tryptophanyl fluorescence at different concentration of ammonium sulfate at pH 7.0 (Fig. 4e). The far UV CD spectra of the PvTRAg33.5 have shown minima at 208 and 222 nm, a characteristic feature of the α-helical structure. Increase in the α-helical structure was observed as salt concentration increased from 0 to 90 mM. Figure 4e (inset) shows the emission spectra of the tryptophanyl residues of the PvTRAg33.5 at different salt concentrations. The increase in fluorescence on addition of the salt indicates opening of the hydrophobic tryptophanyl residues towards the polar solvent. From the far UV CD and fluorescence experiments, we may conclude that increase in salt concentration has disrupted the tertiary structure of the protein without destabilizing its secondary structure.

#### Effect of ethanol

Alcohols can be used as a model for plasma membrane [41]. Some of the tryptophan-rich proteins are found to get anchored to the RBC membrane [4], [5]. We have checked the effect of ethanol on the PvTRAg33.5 by the far UV CD and tryptophanyl fluorescence spectroscopy to study the effect of hydrophobic environment on the protein conformation. Fig. 4f shows stabilization of the secondary and tertiary structure (inset) in the presence of 0 to 40% and 0 to 35% ethanol respectively. But further increase in the alcohol concentration disrupts the tertiary as well as secondary structure.

### Secondary structure and molecular model of the PvTRAg33.5

The secondary structure prediction studies show that the exon 2 encoded polypeptide predominantly comprises of helix and coil regions. A consensus secondary structure prediction sequence was drawn from the results of the different secondary structure prediction servers (Fig. S2). In this, the sequences of each and every amino acid position was assigned either helix or coil or extended sheets only when at least 50% of the above web servers predicted the similar secondary structure. The consensus secondary structure prediction showed that the PvTRAg33.5 has 76.2% helix and 23.8% coil with no extended sheets. This helical content derived from the analysis was more than that of the above mentioned experimental CD data.

In the absence of a clear structural homologue from PDB or pattern prediction, knowledge-based approach to structure prediction employing the fold recognition and protein threading methods was undertaken to determine computationally a suitable template model for the exon 2 encoded polypeptide. The results from the diverse threading servers were compiled and analyzed to derive possible templates based on a three-fold criterion. Models predicted with less than 60% accuracy, comprising short segments of fewer than 90 amino acid residues and structures consisting mainly of β-sheets were not included as these contradicted both experimental and secondary structure consensus data. Several models were built using the templates so obtained (PDB Ids: 2c5k, 1is1, 2a2f, 2lis, 1zro, 2bs5, 2hsb, 2pe4, 2c6j, 1u4q, 2j68 and models from SAM-T08 and iTASSER servers) in the Discovery Studio v1.7. The loops were built using the LOOPREFINEMENT module. A high quality model was selected based on the probability density function (PDF) calculated by the program. The PDF is derived from spatial restraints while building the model and identifies regions of high energy. The final model showed similarity to template models predicted via SAM-T08 and PHYRE. The stereochemistry of the final model was checked with the program PROCHECK [35].

The refinement of the model with special emphasis on the loops was carried out using molecular dynamics simulation. The total energy and simulation temperature were found to remain steady with little fluctuation (Table 2). In addition to this the maximum r.m.s. deviation was observed to be 0.6 Å. Hence, the results from the molecular dynamics simulation indicated that the modeled structure is stable.

**Table 2:** Molecular Dynamics simulation.

Time (ps)	rmsd w.r.t to first conformer (Å)	Potential Energy(kcal/mol)	Temp (K)
0	0	−570.36	303
25	0.56	−566.30	306
50	0.59	−568.31	302
75	0.59	−569.15	301
100	0.60	−570.04	302

## Discussion

For the first time, we describe here the structural characteristic features of a tryptophan- rich antigen (PvTRAg33.5). This protein is not a membrane integrated protein, it lacks cysteine residues, and it is soluble in nature. The tryptophan residues are distributed over the entire length of the mature protein (encoded by exon 2) which may influence the folding of the protein in the absence of the cysteine residues. Thus, the three dimensional conformation of the protein is most probably constrained by the number and hydrophobic nature of these tryptophan residues. The tryptophan residue is conserved in the motifs, however, a great deal of variability is observed for adjacent polar and hydrophobic residues. Despite the absence of orthologues, the profile HMM analysis clearly indicates the presence of similar motifs in the *Plasmodium* species. In non malarial proteins, the tryptophan rich domains have been reported in a variety of the transmembrane surface proteins which play an important role in the folding and assembly [42].

**Figure 5:**
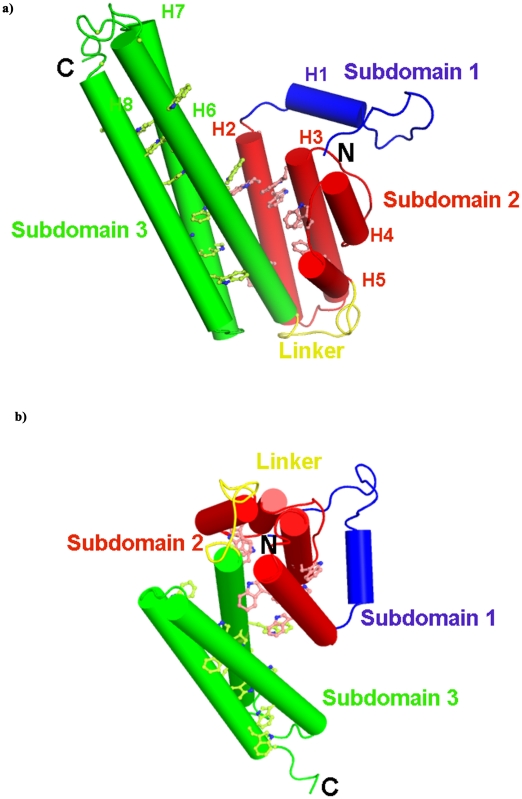
Model of PvTRAg33.5 structure. a) Model of PvTRAg33.5 structure which has three subdmains: subdomain 1 (blue), subdomain 2 (red), subdomain 3 (green) and a linker (yellow) region. The helices are represented as cylinders and the coiled structure as loops. Subdomain 1 adopts a random coil structure except for a two turn helix from residues 16–25 (H1). The second subdomain has 2 long (H2: 29–54 and H3: 60–80) and 2 short helices (H4: 89–98 and H5: 104–113) connected by three loops. The subdomain 3 consists of three long helices (H6: 126–162, H7: 177–213 and H8: 217–244). The tryptophan (Trp) residues in the helices are drawn in ball-and-stick and assigned the helix color. b) The three dimensional model of PvTRAg33.5 indicating the orientation of the subdomains relative to each other at a rotation of 90° to Panel a. The linker region connecting subdomains 2 and 3 is flexible. The tryptophan (Trp) residues in the helices are drawn in ball-and-stick and assigned the helix color.

The modeled tertiary structure of the protein consists of three domains (Fig. 5). The subdomain 1 comprising residues 1–28 adopts a random coil structure except for a two turn helix (H1), the subdomain 2 spanning residues 30–113 has 2 long (H2 and H3) and 2 short helices and the subdomain 3 stretching from residues 126–252 consists of three long helices (H6, H7: and H8). A long loop of 16 residues connects helices 6 and 7 whereas helices 7 and 8 are anti-parallel and are joined by a short loop of 5 residues. The subdomains 2 and 3 are associated by a 12 amino acid long flexible linker region. The linker region adopts a random coil structure and permits different orientations of the subdomains 1 and 2 as regards to the third subdomain. The motifs, M2-M7, identified from MEME have helical structures. The subdomain 3 participates in numerous hydrogen bonds and van der Waals interactions with subdomain 2 while these are limited to the tail residues to subdomain 1.

Thus the PvTRAg33.5 protein belongs to the proteins from the all-alpha class with no identical fold in Structural Classification of Proteins (SCOP) database [43]. The closest structural similar fold in SCOP was observed to be the Duffy binding like domain (DBL) fold which has earlier been observed in *P. knowlesi* (PDB Id: 2C6J) [44] and *P. falciparum* (PDB Ids: 3CML [44] 1ZRO and 1ZRL [45]). The DBL domain possesses two subdomains, each with a three- helical bundle. The subdomain 3 of PvTRAg33.5 has a 3 helix bundle comparable to the DBL domain but a structural superimposition of the long helices of this sub domain revealed that the position of the helices with respect to each other are dissimilar. The main difference is observed in the subdomain 2. A structural superimposition along the long helix H6 of the subdomain 3 indicated that the orientation and positioning of the subdomains 2 and 3 was different with respect to each other in these proteins. These differences indicate that the linker region connecting the subdomain 2 and 3 is flexible and a conformational change can alter their relative orientations.

The CD data indicates that the PvTRAg33.5 has nearly 50% α-helix structure which was also predicted by the secondary structure prediction analysis. However, the protein has shown a total loss of the alpha helical structure if pH was decreased below 4.5. Such structural change from the alpha helical to the beta sheet and vice-versa has been reported in various systems, for example, the beta sheet structure of the HIV's gp120 gets converted into the alpha helix when virus binds to the host membrane protein CD4 [45]–[49]. The PvTRAg33.5 protein, however, remains stable upto pH 4.5 or till 50°C at pH 7.0. Even 40% ethanol could not disrupt its secondary structure. These evidences suggest that the protein is stable enough to withstand some environmental stresses up to certain extent.

The PvTRAg33.5, probably being a small polypeptide, has two state transitions in its secondary structure under the denaturing conditions. The PvTARAg55, probably being a longer polypeptide [12], seems to be more resistant to the effect of low pH than the PvTRAg33.5 (Data not shown). In short, the PvTRAg33.5 is a predominantly α-helical but a loss of the structure occurs under severe environmental stressed conditions. Under stress, either more number of the tryptophan residues gets exposed or the similar number of tryptophan residues exposed with larger extents, as indicated by the tryptophan fluorescence under low pH, high temperature and high concentration of the salt or the non polar solvent. The tryptophan rich regions appear to be critical for the cell-cell fusion activity of the trans-membrane glycoproteins [46]. In nature, certain external stimuli may also cause the structural changes in the proteins leading to the diseased conditions [47]. For example, binding of the ligands could trigger the conformational changes in some of the Trypanosomal enzymes to modulate their activity leading to the disease pathogenesis [48], [49]. The structural perturbation of the PvTRAg33.5 may, therefore, connect with its biological function such as its probable role in the erythrocyte invasion. The Tryptophan residues have also been shown to play an important role in protein-protein interactions and signal transduction [50], [51], [52], [53], [54], [55]. There are rapidly mounting evidence that the proteins must at least partly unfold in order to be translocated across a biological membrane. The precursor proteins are found to unfold before or at the time of their transport across the membranes [56].

In conclusion, for the first time the structural characteristic features of a *Plasmodium* tryptophan-rich protein are described here. The PvTRAg33.5, a novel tryptophan-rich antigen from *P.vivax*, is expressed during the blood stage of the parasite, has a predominantly α-helical structure, and its secondary structure is lost upto 50% under the stressed conditions which may also be faced by the parasite in its host. The modeled structure has three domain architecture and this conformation is stabilized by the presence of a large number of tryptophan residues which in the absence of disulphide bonds influence the folding due to their predominantly hydrophobic nature. Furthermore, the PvTRAg33.5 contains B-cell epitopes which are highly conserved in the parasite population and high antibody response is being generated in humans during *P.vivax* infection. Further studies are required to define the biological function of this protein in the parasite.

## Supporting Information

Figure S1 **Multiple sequence alignment of PvTRAg33.5 (Plasmodb ID: PVX_121897) with close tryptophan rich homologs present in** ***Plasmodium*** **species.** The sequences of these proteins were retrieved from the *Plasmodium* database (www.plasmodb.org) and aligned using ClustalW software at http:/www.ebi.ac.uk/clustalW. The plasmodb ID of *P.vivax* homologs are PVX_090250 for PvTRAg39.8, PVX_109280 for PvTRAg35.2, PVX_090265 for PvTRAg, PVX_101515 for PvTRAg40, PVX_096995 for PvTARAg55, PVX_112655 for PvTRAg80.6 and PVX_101510 for PvATRAg74. The plasmodb ID of *P.falciparum* homologs are PFA0135w for PfMaTrA, PF08_0003 for PfTryThrA, PF10_0026 for TrpA-3, and MAL13P1.269 for LysTrpA. The plasmodb ID of *P.yoelii* homologs are PY06023 for PypAg1, and PY03625 for PypAg3. Stars (“*”) indicate identical amino acids while double (“:”) and single (“.”) dots indicate the conserved and semi-conserved substitutions, respectively. All tryptophan residues are in boldface. The positionally conserved tryptophan residues are shaded grey. Dashes indicate the absence of amino acids. Numbers on the right-hand side indicate the number of amino acid residue. The secondary structure elements for PvTRAg33.5 are indicated on the top of the sequences. The coils are indicated as bold lines and helices as tubes. The helices are numbered H1 to H8. Alignment is according to the PvTRAg33.5 amino acids sequence (complete sequences of all proteins are not shown).
(TIF)

Figure S2 **Secondary structure prediction of exon 2 encoded PvTRAg33.5 by webservers and the consensus secondary structure prediction.**
(TIF)

Supplementary Material S1 Secondary Structure Prediction Servers.
(DOC)
