# Supplementary figures and images for: Plasmodium vivax Tryptophan-Rich Antigen PvTRAg33.5 Contains Alpha Helical Structure and Multidomain Architecture
Source: PLoS One. 2011 Jan 20;6(1):e16294. doi: 10.1371/journal.pone.0016294 (PMC3024423; doi:10.1371/journal.pone.0016294)

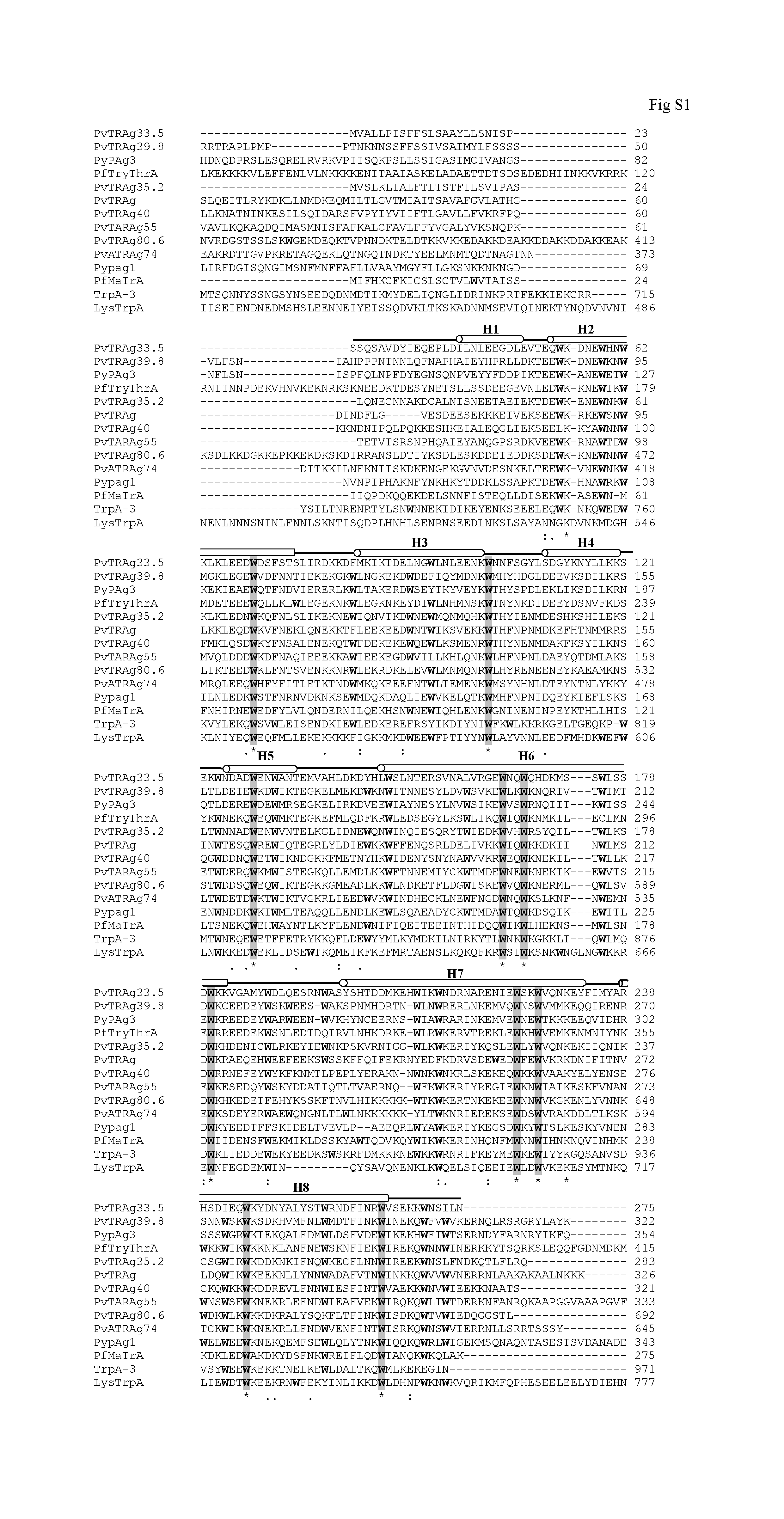

Supplement: Figure S1 — Multiple sequence alignment of PvTRAg33.5 (Plasmodb ID: PVX_121897) with close tryptophan rich homologs present in Plasmodium species. The sequences of these proteins were retrieved from the Plasmodium database (www.plasmodb.org) and aligned using ClustalW software at http:/www.ebi.ac.uk/clustalW. The plasmodb ID of P.vivax homologs are PVX_090250 for PvTRAg39.8, PVX_109280 for PvTRAg35.2, PVX_090265 for PvTRAg, PVX_101515 for PvTRAg40, PVX_096995 for PvTARAg55, PVX_112655 for PvTRAg80.6 and PVX_101510 for PvATRAg74. The plasmodb ID of P.falciparum homologs are PFA0135w for PfMaTrA, PF08_0003 for PfTryThrA, PF10_0026 for TrpA-3, and MAL13P1.269 for LysTrpA. The plasmodb ID of P.yoelii homologs are PY06023 for PypAg1, and PY03625 for PypAg3. Stars (“*”) indicate identical amino acids while double (“:”) and single (“.”) dots indicate the conserved and semi-conserved substitutions, respectively. All tryptophan residues are in boldface. The positionally conserved tryptophan residues are shaded grey. Dashes indicate the absence of amino acids. Numbers on the right-hand side indicate the number of amino acid residue. The secondary structure elements for PvTRAg33.5 are indicated on the top of the sequences. The coils are indicated as bold lines and helices as tubes. The helices are numbered H1 to H8. Alignment is according to the PvTRAg33.5 amino acids sequence (complete sequences of all proteins are not shown). (TIF) [file pone.0016294.s001.tif]
