# Supplementary material for: Plasmodium vivax Tryptophan-Rich Antigen PvTRAg33.5 Contains Alpha Helical Structure and Multidomain Architecture
Source: PLoS One. 2011 Jan 20;6(1):e16294. doi: 10.1371/journal.pone.0016294 (PMC3024423; doi:10.1371/journal.pone.0016294)
Supplement: Supplementary Material S1 — Secondary Structure Prediction Servers. (DOC) [file pone.0016294.s003.doc]

**Supplementary Material**

The prediction servers incorporating different algorithms based on Artificial Neural Network and Hidden Markov Models were used for the secondary structure prediction to minimize the error in prediction. The servers GOR4 [1], Jpred (Jnet, Jpssm, Jhmm) [2,3], SOPMA [4], HNN (Hierarchical neural network), JUFO [5], PSIPRED [6], nnPREDICT [7] and SSPro [8] were used to discern the secondary structure composition of PvTRAg33.5. The consensus secondary structure was derived employing the probability of the predicted sequence and the type of secondary structure prediction from these methods. In the programs where probability prediction was not provided then these predictions were assigned low probabilities. The percentage of secondary structure prediction made was compared with that derived from experimental CD studies.

References

1. Garnier J, Osguthorpe DJ, Robson B (1998) Analysis of the accuracy and implications of simple methods for predicting the secondary structure of globular proteins. J Mol Bio 120: 97-120.

2. Cuff JA, Clamp ME, Siddiqui AS, Finlay M, Barton GJ (1998) JPred: a consensus secondary structure prediction server. Bioinformatics 14: 892-893.

3. Cuff JA, Barton GJ (2000) Application of multiple sequence alignment profiles to improve protein secondary structure prediction. Proteins 40: 502-511.

4. Geourjon C, Deleage G (1995) SOPMA: significant improvements in protein secondary structure prediction by consensus prediction from multiple alignments. Comput Appl Biosci 11: 681-684.

5. Pollastri G, McLysaght A (2005) Porter: a new , accurate server for protein secondary structure prediction. Bioinformatics 21: 1719-1720.

6. McGuffin LJ, Bryson K, Jones DT (2000) The PSIPRED protein structure prediction server. Bioinformatics 16: 404-405.

7. Kneller DG, Cohen FE, Langridge R (1990) Improvements in protein secondary structure prediction by an enhanced neural network. J Mol Biol 214: 171-182.

8. Baldi P, Brunak S, Frasconi P, Soda G, Pollastri G (1999) Exploiting the past and the future in protein secondary structure prediction. Bioinformatics 61: 937-946.
